# Comparative study of *Cronobacter* identification according to phenotyping methods

**DOI:** 10.1186/s12866-016-0768-6

**Published:** 2016-07-11

**Authors:** Emily E. Jackson, Stephen J. Forsythe

**Affiliations:** Pathogen Research Group, School of Science and Technology, Nottingham Trent University, Clifton Lane, Nottingham, NG11 8NS UK

**Keywords:** *Cronobacter*, Phenotyping, Biochemical identification

## Abstract

**Background:**

Microbiological criteria applied to powdered infant formula (PIF) require the absence of all *Cronobacter* spp. Consequently, misidentification of isolates from finished products can lead to significant financial losses for manufacturers and could increase the risk of neonatal infection. Biochemical identification of suspect isolates using commercially available test panels is recommended for use by PIF manufacturers by both the US FDA and ISO standard methods for *Cronobacter* species; however, phenotyping can be unreliable, particularly for a genus such as *Cronobacter* where the taxonomy has been subject to frequent changes. This study compared the predicted identification by commonly used phenotyping kits (API20E and ID32E) for over 240 strains of *Cronobacter* from diverse sources, which had been identified using DNA sequence analysis. In 2015, the databases associated with the API20E and ID32E biochemical test panels were updated, including the recognition of the *Cronobacter* genus. Thus, the identifications from multiple versions the databases were compared to each other and to identifications based on DNA sequencing methods.

**Results:**

Using previous versions of the API20E database, 90.0 % of strains (216/240) resulted in a match for the species identification; however, version 5.0 produced matches for only 82.3 % of strains (237/288). Similarly, the update to version 4.0 in the ID32E database caused the percentage of matches to drop from 88.9 % (240/270) to 43.2 % (139/322). A smaller study showed that the Vitek GN system identified all 14 strains, belonging all seven *Cronobacter* species, as members of the ‘*C. sakazakii* group,’ but also attributed three strains of *Franconibacter helveticus* and *F. pulveris* to this group. *In silco* analysis of a PCR-based method targeting *ompA* predicted that amplification would only occur with *Cronobacter* species and this method may be a feasible alternative to biochemical phenotyping.

**Conclusions:**

These results indicate that commercially available biochemical test panels are not sufficiently reliable for speciation of *Cronobacter* isolates. Although DNA-sequence based methods would be the more reliable approach; however, this is not currently feasible for many food microbiology laboratories. Instead, a previously published PCR-based method targeting *ompA* is suggested as an alternative for identification of *Cronobacter* species based on *in silico* analysis.

**Electronic supplementary material:**

The online version of this article (doi:10.1186/s12866-016-0768-6) contains supplementary material, which is available to authorized users.

## Background

Current microbiological criteria applied to powdered infant formula (PIF) require the absence of all seven *Cronobacter* species in thirty 10 g samples [1]. Subsequently, the misidentification of microorganisms in PIF can lead to significant losses for manufacturers and may present a risk to neonates. The in-house false positive misidentification of an isolate as *Cronobacter* in a batch of product would result in the manufacturer losing productivity and profits. Whereas, a false negative identification, in which a *Cronobacter* isolate is misidentified as a permitted organism, may result in neonatal infections, product recalls, and lost consumer confidence. These losses can be significant for manufacturers as demonstrated in 2011 when a suspected outbreak of *C. sakazakii* in the United States led to product recalls and a subsequent 10 % drop in the manufacturers’ shares [2, 3]. This was despite the lack of laboratory evidence to linking their product to infant infections and deaths [2, 3]. The costs of infection are also significant, due to the long-term effects of the illness, including life-long brain damage. Minor et al. (2015) estimated the cost of *C. sakazakii* infections to be greater than $5 million per case [4].

The *Cronobacter* genus is currently recognized as containing 7 species, whereas prior to 2007 all species within the genus were known as ‘*Enterobacter sakazakii*’. The 2007 and 2008 taxonomic descriptions of the *Cronobacter* genus and its members used biotyping to re-assign *Enterobacter sakazakii* strains to the new species; however, biotyping has been reported to contradict DNA sequence-based phylogeny based on multilocus sequence typing and whole genome sequence analysis [5–8]. While DNA sequence-based methods are considered to be more reliable for identification of *Cronobacter* species, they are also more expensive, more labor intensive, and have a slow turnaround time. Consequently, it is not currently feasible for PIF manufacturers to employ these methods.

Biochemical identification of suspect *Cronobacter* isolates from PIF is often recommended. Previously, the 2006 ISO standard recommended use of the ID32E biochemical test panel, but the proposed new ISO standard specifies traditional microbiological methods for confirmation with seven required biochemical tests [9, 10]. Six of these tests are included in the ID32E test panel and the proposed standard states that such kits can be used in place of more traditional biochemical methods [10]. Additionally, though the FDA Bacteriological Analytical Manual (FDA BAM) includes a real-time PCR screening method, the results must be confirmed culturally [11]. The recommended cultural methods can also be used independently for identification of suspect isolates when PCR-based methods are not available [11]. According to the FDA BAM, biochemical identification of suspect *Cronobacter* isolates should be carried out using the ID32E test kit or Vitek 2 GN cards [11].

Though they are widely used and recommended by both ISO and the FDA, biochemical test panels, like the ID32E, can be unreliable for various reasons. First, their reliance on visual detection of color changes make accurate identification difficult due to subjectivity when reading the test results. Additionally, discrepancies between the different test kits have also been reported. For example, Iversen et al. correctly identified 90 % of *Cronobacter* isolates to the nearest match of ‘*Enterobacter sakazakii*’ with the ID32E test kit, but only 70 % of those isolates were correctly identified with the API20E [12]. Finally, the accuracy of identification databases can be problematic due to changes in bacterial taxonomy which are not reflected promptly. This is a particular problem for the genera *Cronobacter* where a number of taxonomic changes have occurred very quickly and some species are very closely related [13–15]. Both the API20E and ID32E databases persisted in using the pre-2007 designation of ‘*E. sakazakii*’ until 2015. Following the 2015 update, version 5.0 of the API20E database reports a result of ‘*Cronobacter* spp.’ while version 4.0 of the ID32E database purports to identify isolates to the species level within the *Cronobacter* genus.

Despite the reliance on phenotyping methods for identification of *Cronobacter* spp. in PIF, no large scale analysis of the accuracy of such methods has been undertaken. The current study was conducted to compare the species identifications using both old and new versions of the API20E and ID32E databases. The biochemical profiles from over 240 previously identified strains in the culture collection at Nottingham Trent University (NTU) were analysed with the updated databases and the results were compared to the previous identifications. Additionally, a subset of 19 strains were analysed with Vitek GN cards and the results from all three biochemical methods were compared. A PCR probe-based method targeting the gene *ompA* was explored as an alternative method because it requires only a PCR thermocycler and gel electrophoresis capabilities, which are available in most microbiology laboratories. This method was analyzed *in silico* using 223 full genome sequences belonging to *Cronobacter* and related genera.

## Methods

### Biochemical identification

API20E and ID32E profiles and identifications were obtained from the archived records of the NTU culture collection. These profiles were re-analysed using version 5.0 of the API20E database and version 4.0 of the ID32E database. It was not feasible to determine all predicted identifications for recently acquired strains since the older database versions were no longer accessible. This resulted in differences in the number of total strains for some analyses. All strains used in this study have been identified to the species level using 16S rDNA sequencing or sequencing of the *fusA* allele as part of the multilocus sequence typing (MLST) scheme for *Cronobacter* spp. [16, 17]. The strains used in these analyses were isolated from 21 different countries over a period of 65 years (1950–2015). These isolates were obtained from a variety of sources including PIF and PIF manufacturing environments, foods, herbs and spices, and clinical samples.

Due to the use of different versions of the associated databases, the date of the original analysis influences the species identification. For the older versions of both databases, a result of “*Enterobacter sakazakii*” was considered to be a match for all *Cronobacter* species and strains, as this was the closest identification available in the database at the time. A result of “*Cronobacter* spp.” was considered as to be a match for all *Cronobacter* strains with the updated version of the API20E database; however, with the updated ID32E database, a result was only considered to be a match if the identified species matched the species as determined by one or more DNA sequence-based methods. Strains giving a result of “Unacceptable profile” were considered to be mismatches. Identifications with the older versions of the databases were not available for all strains. These results were assigned as “unknown” and were not considered to be either matches or mismatches. The *X*^2^ test was used to determine if the percentage of matches differed significantly between database versions. Test statistics and *p*-values were calculated using Microsoft Excel. A *p*-value of <0.05 was considered to be significant. Only strains which had been designated as matches or mismatches were included in this analysis.

A selection of 19 strains, including 14 C*ronobacter* strains, were additionally analysed with the semi-automated Vitek system; Table 1. Strains were selected for this analysis because their biochemical profiles and corresponding identifications have been published previously [18–20]. The non-*Cronobacter* strains were included in the analysis as they have been previously misidentified as members of the *Cronobacter* genus [20]. The Vitek GN analyses were carried out according to the manufacturer’s instructions using the Vitek 2 Compact machine and GN cards. Species identification was performed using version 05.04 of the Vitek software and version 05.00.011 of the Vitek GN database.

**Table 1:** Bacterial species and strains analysed with the Vitek GN system

Species	Strain	Source	Country of isolation	Year
*Cronobacter condimenti*	LMG26250^T^	Food	Slovakia	2010
*Cronobacter dublinensis*	LMG23823^T^	Environmental	Ireland	2004
*Cronobacter malonaticus*	8	Weaning food	Czech Republic	2004
*Cronobacter muytjensii*	16	Spice	Unknown	2005
*Cronobacter sakazakii*	4	Clinical	Canada	1990
*Cronobacter sakazakii*	5	Clinical	Canada	1990
*Cronobacter sakazakii*	12	Clinical	Czech Republic	2003
*Cronobacter sakazakii*	1436	Food	Turkey	2010
*Cronobacter sakazakii*	1437	Food	Turkey	2010
*Cronobacter sakazakii*	1438	Food	Turkey	2010
*Cronobacter turicensis*	9	Weaning food	U.K.	2003
*Cronobacter turicensis*	LMG23827^T^	Clinical	Switzerland	2005
*Cronobacter universalis*	NCTC9529^T^	Water	U.K.	1956
*Cronobacter universalis*	1435	Food	Turkey	2010
*Escherichia hermanii*	162	Rice	U.K.	2004
*Franconibacter helveticus*	LMG23732^T^	Fruit powder	Switzerland	2007
*Franconibacter pulveris*	1393	Ingredients	U.K.	2011
*Franconibacter pulveris*	LMG24057^T^	Fruit powder	Switzerland	2008
*Siccibacter colletis*	NCTC14934^T^	Food ingredient	U.K.	2011

### *In silico analysis of the* ompA *PCR method*

The BLAST function of the PubMLST *Cronobacter* database was used to extract the *ompA* sequences with 500 nucleotide flanking regions from 187 *Cronobacter* strains representing all seven species [17, 21]. Additionally, the corresponding gene sequences were extracted from 36 strains of closely related organisms, including members of the *Enterobacter*, *Citrobacter*, *Franconibacter*, *Siccibacter*, and *Yersinia* genera. The GenBank accession number for the *ompA* sequence used in the BLAST search is DQ000206 [22].

Extracted sequences were aligned in MEGA 6 and were examined for the presence of primer binding sites for the PCR primers ESSR-F and ESSR-R from the method described by Mohan-Nair and Venkitanarayanan [22, 23].

## Results and discussion

Since previous version(s) of the API20E database identified strains as “*Enterobacter sakazakii*,” this result was considered to be a match for all *Cronobacter* species. A significant difference (*p* < 0.05, *X*^2^ test) was found in the percentage of strains producing a profile that resulted in a match with the different versions of the database. Only 82.3 % of strains resulted in a match with version 5.0, while 90.0 % of strains resulted in a match with previous version(s). These results are summarized in Additional file 1: Table S1. The majority of strains (68.8 %) produced matches with both the old and new versions of the database; however, 6.3 % of strains produced profiles which resulted in match previously but a mismatch following the update to the API20E database. Only one strain (0.4 %) showed a mismatch in the archived records, and a match following the update to the database.

Strains of *Cronobacter* were misidentified as *Enterobacter aerogenes*, *E. amnigenus*, and *E. cloacae* with the previous version of the API20E database and as *E. aerogenes*, *E. amnigenus*, *E. cancerogenus*, *E. cloacae*, and *Serratia liquefaciens* with the updated version. Strains of *E. cloacae* and *E. hormaechei* were misidentified as *Cronobacter* spp. (or ‘*E. sakazakii*’) with both versions of the database. Though most profiles returned a percent identification for each species, seven profiles did not do so with the updated database, returning a result of “Unacceptable profile”. These results were considered to be mismatches and such results incorporate further uncertainty into identification of isolates producing these profiles.

A total of 61 API20E profiles were observed for confirmed strains of *Cronobacter* species. All of these profiles were positive for amygdalin fermentation and negative for hydrogen sulfide production. More than 90 % of these profiles were also positive for β-galactosidase (96.7 %), arginine dihydrolase (93.4 %), citrate utilization (93.4 %), and D-glucose (95.1 %), D-mannitol (91.8 %), L-rhamnose (91.8 %), D-saccharose (93.4 %), D-melibiose (96.7 %), and L-arabinose (93.4 %) fermentation. Additionally, more than 90 % of these profiles were negative for urease activity (95.1 %). The observed reactions for arginine dihydrolase, citrate utilization, hydrogen sulfide production, urease activity, and acid production from glucose, D-mannitol, L-rhamnose, D-saccharose, D-melibiose, and L-arabinose match the original description of the *Cronobacter* genus [5, 6].

Of the 61 API20E profiles, the most common were 3305373, 3307173, and 3305173 which represented 60, 41 and 40 strains, respectively. Together, these three profiles were observed for more than half of the strains analysed (141/260; 54.2 %). These profiles differed from one another by only two traits; gelatinase activity and inositol fermentation. Each of these profiles resulted in a species identification of “*E. sakazakii*” with version 4.0 of the database and were identified as *Cronobacter* spp. with version 5.0. Profile 3307173 was only observed for confirmed strains of *Cronobacter*, belonging to four different species; *C. sakazakii* (*n* = 34), *C. malonaticus* (*n* = 4), *C. turicensis* (*n* = 2), and *C. dublinensis* (*n* = 1). In addition to *C. sakazakii* (*n* = 32) and *C. malonaticus* (*n* = 8), strains of *E. hormaechei* (*n* = 3) and *E. cloacae* (*n* = 1) were identified with profile 3305173. Strains of *C. sakazakii* (*n* = 49), *C. malonaticus* (*n* = 7), *C. turicensis* (*n* = 4) and *E. hormaechei* (*n* = 6) produced profile 3305373. Therefore, while these profiles were frequently observed for *Cronobacter* spp., they are not necessarily specific to members of this genus and may be produced by members of the *Enterobacter* genus.

Though easily distinguished by colony morphology on Druggan-Iversen-Forsythe agar, *Enterobacter* isolates have been misidentified as members of the *Cronobacter* genus in the past. For example, two illnesses in Mexico were attributed to *C. sakazakii* using cultural and molecular identification methods [24]. Subsequent DNA sequence-based analysis of the patient isolates, however, identified them as members of the *Enterobacter* genus [25]. Similarly, Townsend et al. (2008), used 16S rDNA sequencing to identify 10 clinical strains of *Enterobacter hormaechei* that had been previously identified as *C. sakazakii* by phenotyping [26]. The correct identification and genotyping of these isolates revealed that a previously unrecognised noscomial outbreak of *E. hormaechei* had occurred [26]. These investigations show that clinical *Enterobacter* isolates have been mistaken for *C. sakazakii* and though infectious, these organisms are not addressed in the microbiological criteria applied to PIF [1, 25–29]. Although false positive results were observed for *E. cloacae* and *E. hormaechei*, these organisms have been isolated from infant formula and can result in neonatal infections [25, 26]. Even though the the risk from these organisms in PIF has not been closely examined, they may present a hazard and their misidentification as *Cronobacter* spp. could result in rejection of an unsafe batch of formula.

For the ID32E biochemical kit, Fanjat et al. noted that variation in the L-arabinose and α-maltosidase tests resulted in misidentification of some *Cronobacter* isolates (then known as “*E. sakazakii*”) when using version 2.0 of the ID32E database [30]. The update to version 3.0 reflected this variability, resulting in 100 % correct identification of the isolates [30]. Conversely, updating the database to version 4.0 resulted in higher numbers of misidentifications of *Cronobacter* species (*p* < 0.05, *X*^2^ test). With the previous versions of the ID32E database, 88.9 % of strains produced a match (as *E. sakazakii*); however, this percentage drops to only 43.2 % when the updated version of the database is used (Additional file 2: Table S2).

Over one third of strains (37.9 %) produced a match both in the culture collection records and with version 4.0 of the ID32E database; however, 36.6 % of strains produced a match in the culture collection records, but a mismatch after the database was updated. As with the API20E results, only one strain (0.3 %) showed a mismatch in the archived culture collection records, but a match with the updated database.

The major limitation with the update to the ID32E database is that it attempts to identify strains to the species level, but members of the *Cronobacter* genus can be difficult to differentiate without highly specific methods. In particular, *C. sakazakii* and *C. malonaticus* are so closely related that they cannot be reliably differentiated using 16S rDNA sequencing methods [16]. The ID32E biochemical test panel is simply not specific enough to differentiate the seven species of *Cronobacter*. If these results are examined to only the genus level, 82.3 % of confirmed *Cronobacter* strains were assigned to the *Cronobacter* genus using version 4.0 of the ID32E database. Though this still represents a significant difference (*p* < 0.05, *X*^2^ test) in the accuracy of identification from the archived culture collection records, it is considerably more accurate than identification to the species level.

With the ID32E test kit, *Cronobacter* strains were misidentified as *Enterobacter cancerogenus*, *E. cloacae*, and *Stenotrophomonas maltophilia* with the old version(s) of the database and as *Buttiauxella agrestis*, *Citrobacter freundii*, *Cit. koseri*, *E. aerogenes*, *E. cancerogenus*, *E. cloacae*, *Escherichia vulneris*, *Leclercia adecarboxylata*, *Serratia liquefaciens*, *S. rubidea*, and *Stenotrophomonas maltophilia* with the updated database. Meanwhile, strains of *Cit. freundii*, *Cit. koseri*, *E. hormaechei*, *Escherichia hermanii*, *Franconibacter helveticus*, *F. pulveris*, *Leclercia adecarboxylata*, *Pantoea* spp., *Siccibacter colletis*, and *Sic. turicensis* were misidentified as *Cronobacter* species with the previous version of the database while *Cit. freundii*, *Cit. koseri*, *E. hormaechei*, *E. pyrinus*, *Esh. hermanii*, *F. helveticus*, *L. adecarboxylata*, and *Siccibacter turicensis* were misidentified as *Cronobacter* species with the updated version of the database. Misidentification of *Enterobacter* strains as *Cronobacter* spp. also occurred with the API20E test kit and the implications of such misidentifications were discussed previously. The misidentifications of *Franconibacter* and *Siccibacter* species as members of the *Cronobacter* genus is unsurprising as these species were briefly considered to be part of the *Cronobacter* genus [13–15, 20]. Forty-eight ID32E profiles returned a species identification with a description of ‘”Unacceptable profile” instead of a percent identification with the updated version of the database. As with the API20E profiles, this produces more uncertainty in the species identification resulting from those profiles.

A total of 155 ID32E profiles were observed for confirmed strains of *Cronobacter* species. Nearly all (152/155, 98.1 %) of the profiles give a positive result for β-glucuronidase activity. Similarly, only one profile (0.7 %) has a positive reaction for rhamnose acidification. More than 90 % of the profiles for known *Cronobacter* spp. produced positive results for ornithine decarboxylase (90.3 %), arginine dihydrolase (94.2 %), β-glucosidase (98.1 %), malonate utilization (95.5 %), and acidification of maltose (91.0 %), D-glucose (91.0 %), D-saccharose (97.2 %), L-arabinose (93.6 %) and D-arabitol (90.3 %). Additionally, more than 90 % of these profiles were negative of L-aspartic acid arylamidase (97.4 %), α-galactosidase (92.3 %), and acidification of both L-arabitol (97.4 %), and 5-ketogluconate (94.3 %). The observed reactions for ornithine decarboxyase, arginine hydrolase, maltose utilization and acidification of D-glucose and L-arabinose match the original genus description of *Cronobacter*; however, the results for the acidification of L-rhamnose and D-arabitol do not match the original description of the genus [5, 6]. These differences may be due to the use of different phenotyping methods when defining the genus. Additionally, the results for 4-nitrophenyl-α-D-glucopyranoside, and acidification of D-arabitol and D-sorbitol differ significantly from the expected phenotypes specified in the proposed ISO standard [10]. While only 58.7 % of *Cronobacter* ID32E profiles were positive for 4-nitrophenyl-α-D-glucopyranoside, the ISO standard states that 100 % of strains produced a positive result for this test [10]. Similarly, 74.2 % of ID32E profiles were found to be positive for the acidification of D-sorbitol; however, none of the strains included in the ISO standard produced a positive result [10]. Finally, nearly all of the profiles (90.3 %) were positive for the acidification of D-arabitol, but only a few strains of *C. dublinensis*, *C. turicensis*, and *C. universalis* were reported to produce positive results in the ISO standard [10]. The presence of such discrepancies suggests that the biochemical test panel may not be completely accurate in its characterization of isolates. Importantly, biochemical test panels are suggested as an alternative to more traditional biochemical test methods in the draft ISO standard; however, the differences between the expected results given in the standard and the results reported here suggest that the ID32E test kit is not an acceptable alternative method for biochemical identification of suspect *Cronobacter* isolates [10].

The most common ID32E profile was 34276767250 (*n* = 34). Only *C. sakazakii* strains produced this profile, indicating a match to the species level. In addition to the tests matching the genus description given above, this profile is also positive for palatinose acidification, a characteristic given in the original description of *C. sakazakii* [6]. The remaining characteristics used for the description of the species are not included in the ID32E test kit. The next two most common profiles were 34276767050 (*n* = 22) and 34276763050 (*n* = 19). Each of these profiles was observed for three *Cronobacter* species. Profile 34276767050 was produced by strains of *C. sakazakii* (*n* = 16), *C. malonaticus* (*n* = 4), and *C. turicensis* (*n* = 2). According to the updated ID32E database (version 4.0), these strains were all identified as *C. sakazakii*; however this result is a match for only 16/22 strains (72.3 %). Similarly, profile 34276763050 was observed for *C. sakazakii* (*n* = 16), *C. malonaticus* (*n* = 2), and *C. turicensis* (*n* = 1). The species identification for this profile reported by the updated database was *C. dublinensis*. Though correct to the genus level, none of the strains producing this profile resulted in a match to the species level. It should also be noted that these three most common profiles differ from one another in only two tests, α-glucosidase activity and trehalose acidification.

Both the API20E and ID32E biochemical kits include a test for inositol fermentation. Of the 61 API20E profiles, 62.3 % were positive for inositol fermentation. A total of 159 strains were identified as being inositol positive with this test kit, including 112 strains of *C. sakazakii*. With the ID32E test kit 80.7 % of the 155 unique profiles were positive for inositol fermentation. Of the 280 strains identified as being inositol positive with the ID32E test kit, 192 were *C. sakazakii*. As a major pathogenic species in the genus, the high number of *C. sakazakii* strains showing positive results for inositol fermentation is interesting due to the association of the trait with virulence [31]; however, this result may be due to over representation of clinical isolates of *C. sakazakii* in the culture collection and further investigation may be necessary for confirmation.

Two of the major limitations of the API20E and ID32E kits are that they rely on a limited number of tests (20 or 32, respectively) and are subjectively based on visual detection of a color change when reporting results. Thus, the interpretation of the colors by an individual researcher could affect the reported profile and subsequent species identification. The Vitek system attempts to avoid these problems using 64 biochemical tests and automated detection of the color changes for more consistent results. Using a subset of strains, the Vitek system identified all 14 *Cronobacter* strains as members of the “*Cronobacter sakazakii* group”; Table 2. Although this term has no taxonomic standing and has not been defined, it was taken here as being equivalent to the *Cronobacter* genus. In contrast, the API20E misidentified 8/14 (57.1 %) of these strains to the genus level and the ID32E misidentified 13/14 (92.9 %) to the species level. Notably, though they were assigned to the ‘*C. sakazakii* group’ with the Vitek GN card, the type strains for *C. condimenti* (LMG26250^T^), *C. turicensis* (LMG23827^T^), *C. universalis* (NCTC9529^T^) and *C. dublinensis* (LMG23823^T^) were misidentified with both the API20E and ID32E test kits; Table 2. The ID32E kit did identify these strains as members of the *Cronobacter* genus, but did not correctly identify these type strains to species level.

**Table 2:** Comparison of the API20E, ID32E, and Vitek GN methods for identification of *Cronobacter* and closely related species

		Vitek GN	API20E		ID32E	
Strain	Species	Identification	Profile	Species identification (v5.0)	Profile	Species identification (V4.0)
LMG26250^T^	*Cronobacter condimenti*	*Cronobacter sakazakii* group	3367373	*Cronobacter spp*.	34217360051	*Cronobacter muytjensii*
LMG23823^T^	*Cronobacter. dublinensis*	*Cronobacter sakazakii* group	7347373	*Cronobacter spp*.	34256166211	*Cronobacter malonaticus*
8	*Cronobacter malonaticus*	*Cronobacter sakazakii* group	3004153	*Escherichia vulneris*	34774563051	*Cronobacter malonaticus*
16	*Cronobacter muytjensii*	*Cronobacter sakazakii* group	0004153	*Serratia plymuthica*	30676563051	*Cronobacter malonaticus*
4	*Cronobacter sakazakii*	*Cronobacter sakazakii* group	3305173	*Pantoea spp. 3*	34074743011	*Enterobacter cloacae*
5	*Cronobacter sakazakii*	*Cronobacter sakazakii* group	3305173	*Pantoea spp. 4*	14074743211	*Enterobacter amnigenus*
12	*Cronobacter sakazakii*	*Cronobacter sakazakii* group	0005173	*Escherichia vulneris*	30674773050	*Cronobacter malonaticus*
1436	*Cronobacter sakazakii*	*Cronobacter sakazakii* group	3305373	*Cronobacter spp*.	34276763011	*Cronobacter malonaticus*
1437	*Cronobacter sakazakii*	*Cronobacter sakazakii* group	3305173	*Cronobacter spp*.	34276763010	*Cronobacter malonaticus*
1438	*Cronobacter sakazakii*	*Cronobacter sakazakii* group	1304373	*Enterobacter gergoviae*	14234767010	*Cronobacter malonaticus*
9	*Cronobacter turicensis*	*Cronobacter sakazakii* group	1006523	*Cronobacter spp*.	30074773050	*Enterobacter cloacae*
LMG23827^T^	*Cronobacter turicensis*	*Cronobacter sakazakii* group	7315373	*Enterobacter gergoviae*	34276767211	*Cronobacter malonaticus*
NCTC9529^T^	*Cronobacter universalis*	*Cronobacter sakazakii* group	3205373	*Escherichia vulneris*	24276777051	*Cronobacter malonaticus*
1435	*Cronobacter universalis*	*Cronobacter sakazakii* group	3304373	*Cronobacter spp*.	34356767010	*Cronobacter sakazakii*
162	*Escherichia hermanii*	*Pantoea* spp.	1004153	*Cronobacter spp*.	34676767050	*Cronobacter sakazakii*
LMG23732^T^	*Franconibacter helveticus*	*Cronobacter sakazakii* group	1024153	*Cronobacter spp*.	30675567010	*Cronobacter sakazakii*
1393	*Franconibacter pulveris*	*Cronobacter sakazakii* group	3004173	*Cronobacter spp*.	04275763310	*Enterobacter cloacae*
LMG24057^T^	*Franconibacter pulveris*	*Cronobacter sakazakii* group	3004173	*Escherichia vulneris*	04275773310	*Enterobacter cloacae*
NCTC14934^T^	*Siccibacter colletis*	*Pantoea* spp.	3204153	*Escherichia vulneris*	04077563310	*Buttauxiella agrestis*

In addition, the Vitek GN card misidentified members of the *Franconibacter* genus as members of the “*C. sakazakii* group”. The type strain of *F. helveticus* (LMG23732^T^) was also identified as a member of the *Cronobacter* genus by the other two test kits, while the type strain of *F. pulveris* (LMG24057^T^) was misidentified with the API20E test panel; Table 2. As mentioned previously, this not unexpected as current strains in the *Franconibacter* genus were briefly considered to be a part of the *Cronobacter* genus before *Franconibacter* was defined [13, 15]. The false positive results generated by the *Franconibacter* species with the Vitek system are important for PIF manufacturers, as these species have not been linked to any cases of human illness but occur in similar ecosystems as *Cronobacter* species [15]. The international microbiological criteria applied to PIF require the absence of all *Cronobacter* species and this misidentification may cause manufacturers to incorrectly reject a batch of microbiologically safe infant formula.

Of the three biochemical methods evaluated in this study, the Vitek system performed better than the API20E or ID32E for identification of a subset of strains, including the type strains for all *Cronobacter* species and strains which had been previously misidentified. Though some false positive results were observed, this method was able to identify all tested *Cronobacter* species as members of the “*Cronobacter sakazakii* group;” however, relatively few strains were analysed with this method and analysis of a larger number of strains would be necessary to properly determine the accuracy of the Vitek system for identification of *Cronobacter* isolates. The FDA BAM method does suggest using either the Vitek 2 GN cards or the ID32E biochemical test panel for identification of suspect isolates, but there is a high cost associated with the initial purchase of the Vitek 2 instrument. Thus, while this method did seem to perform better in this small-scale analysis, the technology may unfortunately not be accessible to all PIF manufacturers and food testing laboratories.

As reported here and by others, commercially available biochemical test panels are not sufficient for accurate identification of *Cronobacter* species and even show difficulties in identifying strains to the genus level [7, 20, 30, 32]. Thus, the use of an alternative method may be necessary for accurate species identification of suspect *Cronobacter* isolates from PIF. DNA sequence-based identification methods are more reliable, but also more expensive, labor-intensive and have a long turnaround time. As DNA sequencing becomes cheaper and easier, it may be possible to incorporate it into manufacturers’ testing schemes, but this is not currently feasible. Similarly, while many other alternative methods are available for identification of *Cronobacter* spp., they may not be accessible to all laboratories. For example, real-time PCR methods and MALDI-TOF MS can be used to identify suspect isolates; however, these methods require expensive, specialized equipment and trained personnel that will prevent some laboratories from utilizing such techniques.

Several PCR probe-based identification methods have been proposed for the *Cronobacter* genus, which may provide an alternative to biochemical phenotyping or DNA sequence-based identification [22, 33–39]. These methods do not require any specialized equipment beyond a PCR thermocycler and gel electrophoresis capabilities, which would be expected in most, if not all, microbiology testing laboratories. Previous analysis comparing PCR probes with *Cronobacter* genome sequences showed the methods targeting the genes *ompA* and *rpoB* were most likely to result in accurate identification [20, 22, 38, 39]. The *rpoB* method requires a separate PCR primer pair and different amplification conditions for each of the seven *Cronobacter* species [38, 39]. In contrast, the *ompA* method only requires one primer pair and therefore is less laborious and has a shorter turnaround time [22]. Though the PCR method targeting *ompA* was developed to identify “*E. sakazakii*,” strains previously known by this name were reclassified as members of the *Cronobacter* genus [5, 6]. The analysis of Jackson et al. showed that the method targeting *ompA* was able to identify the type strains of all seven *Cronobacter* species, while excluding closely related strains, particularly those belonging to the *Franconibacter* and *Siccibacter* genera [20, 22].

For a thorough examination of the *ompA* PCR method, *in silico* analysis of full genome sequences was used. This method enabled the analysis of a large cohort of internationally derived strains that are not centrally available. To determine if this method could be used for identification of *Cronobacter* from PIF, the genomes of 187 *Cronobacter* and 36 non-*Cronobacter* strains were examined for the presence of the primer sequences targeting *ompA*. Table 3 shows the number of mismatches with each primer and the expected amplicon size for each species. The ESSR-R primer showed a higher number of mismatches than the ESSR-F primer for non-*Cronobacter* species and all *F. helveticus* strains showed 7 mismatches with the ESSR-R primer. Four of these strains were used in the previously reported laboratory evaluation of this method and none produced amplicons [20]. It was, therefore, concluded that strains with 7 or more mismatches to the ESSR-R primer would not produce amplicons with this method.

**Table 3:** Predicted PCR amplification of *ompA* locus

		# mismatches		
Species	n	ESSR-F^a^	ESSR-R	Predicted amplicon size (bp)^b^
		(22 bp)	(19 bp)	
*Cronobacter sakazakii*	101	0	1–2	468
*Cronobacter malonaticus*	45	0	2	468
*Cronobacter dublinensis*	14	0	2	469
*Cronobacter turicensis*	10	0	2	468
*Cronobacter muytjensi*	9	0	0	469
*Cronobacter universalis*	7	0	2	468
*Cronobacter condimenti*	1	0	2	469
*Franconibacter pulveris*	7	1	8	NA
*Franconibacter helveticus*	5	2	7	NA
*Siccibacter turicensis*	4	4	10	NA
*Enterobacter asburiae*	2	4–6	8–11	NA
*Enterobacter hormaechei*	2	6–7	8	NA
*Enterobacter* spp.	2	5–6	7	NA
*Citrobacter amalonaticus*	1	7	9	NA
*Citrobacter freundii*	1	6	12	NA
*Citrobacter koseri*	1	7	13	NA
*Citrobacter rodentium*	1	7	13	NA
*Citrobacter youngae*	1	6	13	NA
*Enterobacter aerogenes*	1	5	13	NA
*Enterobacter cancerogenus*	1	5	7	NA
*Enterobacter cloacae*	1	7	8	NA
*Enterobacter ludwiggi*	1	5	7	NA
*Enterobacter massilensis*	1	5	10	NA
*Enterobacter mori*	1	5	7	NA
*Enterobacter sacchari*	1	7	15	NA
*Siccibacter colletis*	1	4	10	NA
*Yersinia regensburgii*	1	5	7	NA

^a^Primer sequences described previously [22]

^b^Expected amplicon size: 469 bp [22]

Based on the *in silico* analysis of the *ompA* gene, none of the non-*Cronobacter* species examined would be expected to produce amplicons with this method. On the other hand, all *Cronobacter* spp. were predicted to produce amplicons of approximately the expected size. Mohan-Nair and Venkitanarayanan reported an expected size of 469 bp, while the *in silico* analysis predicted amplicons of 468-469 bp [22]. This *in silico* analysis indicates that this method should be sufficient to identify all *Cronobacter* species, while excluding members of closely related genera. Though further identification would be needed to identify the isolates to the species level, this would not be necessary for PIF manufacturers to ensure exclusion of all members of the *Cronobacter* genus from their products [1, 27–29]. As this method is capable of identifying all *Cronobacter* spp., it could be used in place of the biochemical test panels for more accurate genus-level identification of suspect *Cronobacter* isolates from PIF.

## Conclusions

Commercially available biochemical test panels, such as the API20E and ID32E, are not sufficient to identify *Cronobacter* isolates at the species level and reliance on these methods will result in false positive and false negative identifications. Only about 80 % of *Cronobacter* strains were correctly identified to the genus level with current versions of the databases associated with either the AP20E or ID32E test kits. Identification to the species level with the ID32E kit resulted in a match for fewer than half of the strains. Though the Vitek GN cards identified all *Cronobacter* strains as members of the ‘*C. sakazakii* group,’ members of the *Franconibacter* were also assigned to this group. In contrast, *in silico* analysis of the PCR probe-based method targeting *ompA* predicted this method could accurately and specifically identify members of the *Cronobacter* genus. While it cannot identify the individual species, this method would be sufficient for manufacturers to ensure the absence of all *Cronobacter* species in PIF as specified by international microbiological criteria. DNA sequence-based methods are more reliable for species identification, but are less practical for PIF manufacturers. Until DNA sequence-based identification is more feasible for routine microbiological testing laboratories, *Cronobacter* species can be identified using a combination of cultural, biochemical and/or molecular methods; however such methods are not applicable for microbial source tracking and epidemiological purposes. Nevertheless, correct genus level identification of suspect *Cronobacter* isolates will ensure that safe products reach consumers and will minimize productivity losses for PIF manufacturers.

## Additional files

Additional file 1: Table S1.
*Cronobacter* spp. identifications using previous and current versions of the API20E database. (XLSX 12 kb) Additional file 2: Table S2.
*Cronobacter* spp. identifications using previous and current versions of the ID32E database. (XLSX 14 kb)
